# Suicide gene therapy using allogeneic adipose tissue-derived mesenchymal stem cell gene delivery vehicles in recurrent glioblastoma multiforme: a first-in-human, dose-escalation, phase I clinical trial

**DOI:** 10.1186/s12967-023-04213-4

**Published:** 2023-05-27

**Authors:** Saeed Oraee-Yazdani, Roozbeh Tavanaei, Fatemeh Rostami, Atieh Hajarizadeh, Marzieh Mehrabadi, Mohammadhosein Akhlaghpasand, Mona Tamaddon, Samin Khannejad, Kaveh Oraii Yazdani, Alireza Zali

**Affiliations:** 1 Functional Neurosurgery Research Center, Shohada Tajrish Comprehensive Neurosurgical Center of Excellence, Shahid Beheshti University of Medical Sciences, PO box: 1988873554, Tehran, Iran; 2 Stem Cell Technology Research Center (STRC), Iran university of medical science (IUMS), Tehran, Iran; 3 Department of Cell and Molecular Biology, School of Biology, College of Science, University of Tehran, Tehran, Iran; 4 Chronic Diseases Research Center, Endocrinology and Metabolism Population Sciences Institute, Tehran University of Medical Sciences, Tehran, Iran; 5 Department of cardiovascular diseases, Zahedan university of medical science, Zahedan, Iran

**Keywords:** Glioblastoma multiforme, Genetic therapy, Mesenchymal stem cells, Mesenchymal stem cell transplantation, Clinical trial

## Abstract

**Background:**

Glioblastoma multiforme (GBM) is associated with remarkably poor prognosis, and its treatment is challenging. This investigation aimed to evaluate the safety of suicide gene therapy using allogeneic adipose tissue-derived mesenchymal stem cells (ADSCs) carrying herpes simplex virus-thymidine kinase (HSV-TK) gene for the first time in patients with recurrent GBM.

**Methods:**

This study was a first-in-human, open-label, single-arm, phase I clinical trial with a classic 3 + 3 dose escalation design. Patients who did not undergo surgery for their recurrence were included and received this gene therapy protocol. Patients received the intratumoral stereotactic injection of ADSCs according to the assigned dose followed by prodrug administration for 14 days. The first dosing cohort (n = 3) received 2.5 × 10^5^ ADSCs; the second dosing cohort (n = 3) received 5 × 10^5^ ADSCs; the third dosing cohort (n = 6) received 10 × 10^5^ ADSCs. The primary outcome measure was the safety profile of the intervention.

**Results:**

A total of 12 patients with recurrent GBM were recruited. The median follow-up was 16 (IQR, 14-18.5) months. This gene therapy protocol was safe and well tolerated. During the study period, eleven (91.7%) patients showed tumor progression, and nine (75.0%) died. The median overall survival (OS) was 16.0 months (95% CI 14.3–17.7) and the median progression-free survival (PFS) was 11.0 months (95% CI 8.3–13.7). A total of 8 and 4 patients showed partial response and stable disease, respectively. Moreover, significant changes were observed in volumetric analysis, peripheral blood cell counts, and cytokine profile.

**Conclusions:**

The present clinical trial, for the first time, showed that suicide gene therapy using allogeneic ADSCs carrying the HSV-TK gene is safe in patients with recurrent GBM. Future phase II/III clinical trials with multiple arms are warranted to validate our findings and further investigate the efficacy of this protocol compared with standard therapy alone.

*Trial registration*: Iranian Registry of Clinical Trials (IRCT), IRCT20200502047277N2. Registered 8 October 2020, https://www.irct.ir/.

## Background

Despite various improvements, the treatment of glioblastoma multiforme (GBM) as the most common primary malignant brain tumor in adults remains a challenge. Given the highly aggressive nature of the GBM, current therapy, including radical resection, radiation, and chemotherapy, could improve the patient survival merely to a limited extent, and almost all patients show recurrence with a 5-year survival rate of 6.8% [1, 2]. There exists no uniformly agreed-upon treatment strategy for recurrent GBM. Based on different individual factors such as previous treatment and performance status, each repeated surgery, radiation, and chemotherapy might be utilized with limited survival benefit. Hence, there is an urgent need for further novel therapeutic strategies to be translated into clinical practice.

Gene therapy has gained popularity as a potential therapeutic modality for GBM during recent decades. In this regard, various approaches have been investigated in prior preclinical studies, including suicide gene therapy, immunomodulatory gene therapy, tumor-suppressor gene therapy, and oncolytic viral therapy [3]. Moreover, for gene delivery, different types of vectors, including viral and non-viral have been evaluated previously. Among the latter, recently, stem cells have received much attention due mainly to a variety of reasons. This involves tumor-tropic migration and homing capacity, as well as their versatile ability to express numerous therapeutic molecules through different engineering methods [4]. Further, according to prior findings, stem cells could exert immunomodulatory effects inside the tumor niche [5]. Thus, regarding the best stem cell type for gene therapy in GBM, one of the best options is mesenchymal stem cell (MSC) based on prior studies [6–8]. MSCs are poorly immunogenic and could be easily obtained from several sources. Adipose tissue-derived MSCs (ADSCs), in particular, could be a potentially suitable carrier for gene therapy in GBM given their high abundance and ease of isolation in addition to all the aforementioned capabilities [6–8]. Additionally, preclinical studies have suggested a significant cytotoxic efficacy for suicide gene therapy using ADSCs expressing herpes simplex virus thymidine kinase (HSV-TK) gene in animal models of GBM [7–11].

Suicide gene therapy using the engineered ADSCs mainly consists of delivering the HSV-TK to the tumor microenvironment and administration of a prodrug, such as ganciclovir. Following the use of ganciclovir, the HSV-TK phosphorylates it and produces a cytotoxic metabolite, which in turn could inhibit the DNA polymerase and result in cell death. After the production of phosphorylated toxic metabolites, they could be transferred from the ADSCs expressing the HSV-TK to the tumor cells and exert a cytotoxic impact on them through various potential pathways, such as gap junctions, apoptotic vesicles, paracrine immunostimulation, and autophagy inhibition [12–16]. This phenomenon, also known as the “bystander effect,” seems to be the fundamental mechanism behind suicide gene therapy. Furthermore, prior preclinical evidence suggests that MSCs show a specific perivascular pattern of migration inside the tumor microenvironment, which is similar to pericytes and results in their integration into the tumor microvasculature [7–11]. Therefore, based mainly on the “bystander effect” and ADSC migratory and homing capacities, we designed a protocol that included administration of prodrug 3 days following the intracerebral injection of ADSCs in order to provide enough time for cell homing and migration.

Clinical evidence concerning the use of stem cells in gene therapy for GBM is significantly limited. Our previous study has been the only one thus far to evaluate the safety and feasibility of suicide gene therapy using autologous bone marrow-derived MSCs (BMSCs) carrying the HSV-TK gene in patients with newly diagnosed GBM [17]. However, to date, no clinical report has assessed the safety and efficacy of using allogeneic ADSCs as gene carriers for suicide gene therapy in the specific population of patients with recurrent GBM. Hence, the present phase I clinical trial aimed to evaluate the safety and feasibility of this newly developed protocol for HSV-TK-mediated suicide gene therapy using ADSC gene vehicle in patients with recurrent GBM for the first time.

## Methods

### Study design and participants

This investigation was an open-label, non-randomized, single-arm phase I clinical trial with a 3 + 3 dose escalation design, which was approved by the institutional review board of the Shohada Tajrish hospital and the ethics committee of Shahid Beheshti University of Medical Sciences (IR.SBMU.REC.1396.224). This clinical trial was also registered at the Iranian Registry of Clinical Trials (IRCT20200502047277N2). In the present study, all patients who were referred to the neurosurgery department of Shohada Tajrish hospital due to recurrent GBM between October 2020 to March 2021 were screened for eligibility. Before enrollment, patients were fully informed regarding the experimental nature of the study intervention, all potential outcomes, and possible complications or intervention-related adverse events (AEs). After that, written informed consent, including all the study procedures was obtained from all patients. To improve the reporting quality, this clinical trial followed Consolidated Standards of Reporting Trials (CONSORT) guidelines.

The inclusion criteria were age ≥ 18 years, Karnofsky performance scale ≥ 70, previous diagnosis of GBM based on histopathological examination of the tumor specimen, recurrent GBM with clinical and radiological evidence highly suggestive of true progression (exclusion of pseudoprogression as mentioned later in this section), complete course of standard treatment for GBM previously, including standard chemoradiotherapy regimen with or without surgical resection, and tumor location accessible for stereotactic cell injection. Exclusion criteria were surgical resection for recurrent GBM, inaccessible tumor location (e.g., brainstem), high suspicion of pseudoprogression (mentioned later in this section), abnormal renal (serum creatinine > 1.5 mg/dL), hepatic (serum bilirubin > 1.5 mg/dL or serum transaminases level > 3 x upper limit of normal), immunologic (known immunosuppression or white blood cell < 3 × 10^3^ cells/µL or neutrophil < 2 × 10^3^ cells/µL), and coagulation profile (INR > 1.2 or platelet < 100,000 cells/µL), active systemic infection or positive tests for human immunodeficiency virus, hepatitis B and C viruses, any prior history of drug hypersensitivity following the use of ganciclovir, pregnancy, any contraindication for magnetic resonance (MR) imaging, and participation in another clinical trial for GBM treatment simultaneously.

Moreover, in order to exclude cases with pseudoprogression, the clinical and radiological characteristics of the patient were taken into account. Regarding this, any case with increased contrast-enhanced tumor (CET) volume, who also had clinical deterioration, increased steroid intake, and mutation status associated with poor prognosis, was included in this study. However, in case of unremarkable clinical status and absence of mutations associated with poor prognosis, further imaging studies were utilized. In this study, radiological evidence suggestive of true progression was considered as apparent diffusion coefficient ratio < 1.5 on diffusion MR imaging, relative cerebral blood volume < 3.0 on perfusion MR imaging, or choline/N-acetylaspartate > 2 on magnetic resonance spectroscopy.

After recruitment, all patients were assigned to the single study arm and no blinding was performed given the goal of the trial, which was safety evaluation. This investigation was performed according to the Declaration of Helsinki and good clinical practice guidelines. All procedures for the preparation of stem cell vectors in this study, such as cell culture and vector transduction, were performed in a good manufacturing practice (GMP)- and clinical-grade clean room, which was specifically designed to produce cellular therapy products. Moreover, all the materials used during the vector preparation process were GMP-grade in order to prevent potential contamination. Quality control at the end of the production process was performed and ADSC vectors were controlled for any potential contamination before their use.

### Cellular vector preparation

For ADSC isolation, 100 mL of subcutaneous adipose tissue was obtained from a healthy donor through lipoaspiration techniques, under sterile conditions. The isolated adipose tissue was then washed with a solution containing warm PBS and antibiotics. After that, using a blade, adipose tissue was cut into 1 mm pieces and mixed with equal volumes of 0.2% collagenase enzyme for 15–30 min in the incubator (95% humidity and 5% CO_2_ at 37 °C). To neutralize the enzyme, the mixture was washed with 10% FBS and centrifuged at 2000 rpm for 15 min. Thereafter by using a 70 μm filter, the stromal vascular fraction was filtered. The number of mononuclear cells was then counted, and in a T-150 flask with DMEM-F12 medium and 10% FBS, 10^6^ cells were seeded. Incubation was performed in the standard condition (95% humidity and 5% CO2 at 37 °C) for 3 days, and the supernatant was transferred to a new flask afterward. This process was performed every 4 days for 3 weeks. After that, to confirm the cell types, flow cytometry was used in each passage to evaluate the surface CD markers, including CD105, CD90, CD73, CD45, and CD34. Furthermore, the differentiation capacity of cells to osteogenic and adipogenic lineages was assessed [17].

**Fig. 1:**
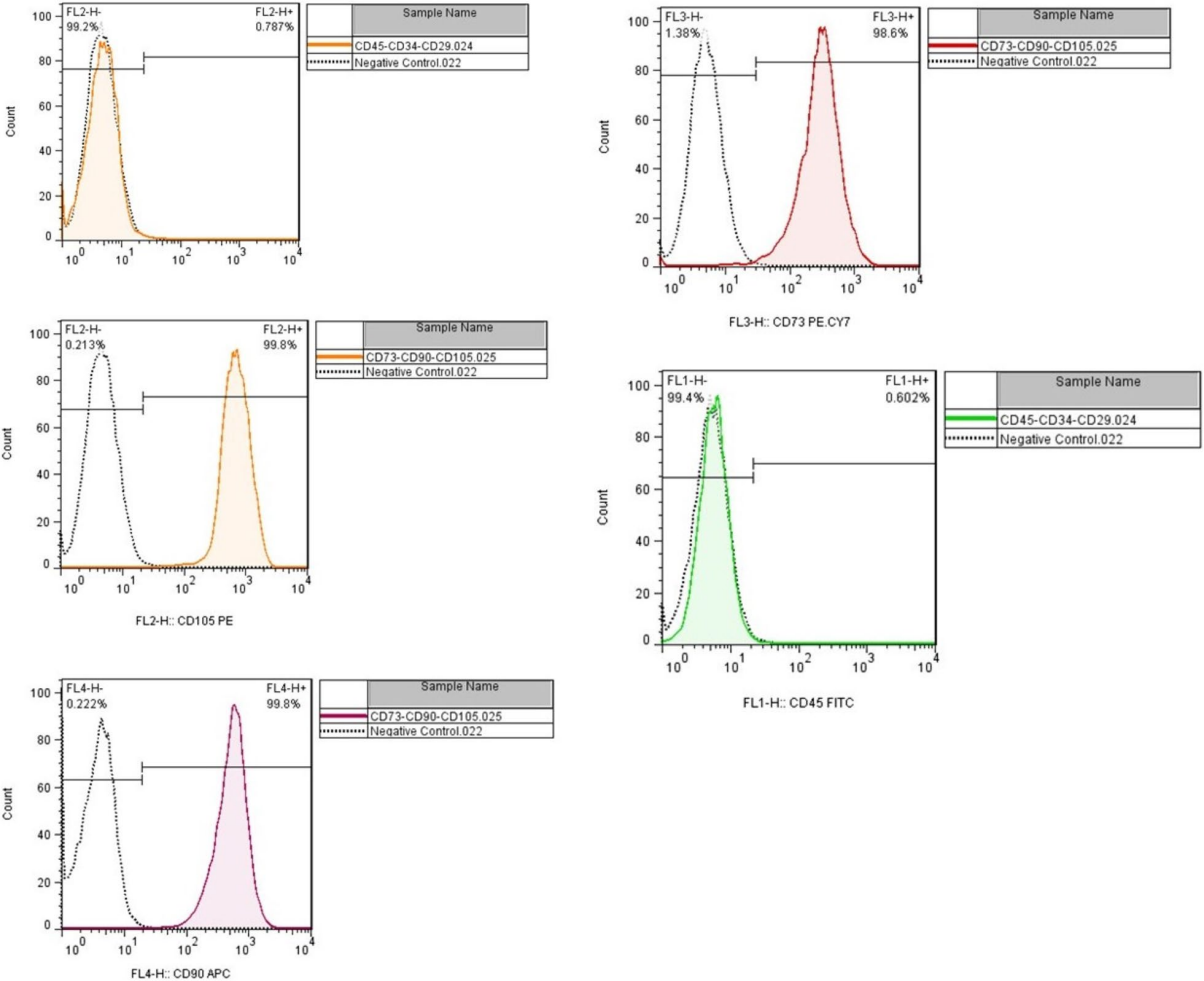
Flow cytometry results demonstrating positive CD73, CD90, CD105, and negative CD34 and CD45 in mesenchymal stem cell (MSC) population

For lentivirus production, the human embryonic kidney (HEK) 293 cell line was cultured in a high-glucose DMEM medium, 10% FBS, 1% nonessential amino acids, and L-glutamine under the standard condition. The gain of function study was performed using a plasmid (pCDH-CMV-MCS-EF1-copGFP), which had been previously digested by BamHI and EcoRI endonuclease enzymes. The TK gene was cloned into the plasmid and the protocol was tested in vitro using the GFP product. After that, by using the calcium phosphate transfection method, the HEK 293 cells (packaging cell line) with 70% confluency were transfected with 21 µg PCDH-TK, 21 µg PsPAX2 plasmid (containing Gag, Pol, Rev, and Tat), and 10.5 µg PMD2 plasmid (containing vesicular stomatitis virus glycol protein) in T75 flask using four optimized reagents containing HBSS 2 × (0.05 M HEPES, 0.28 M NaCl, 0.75 mM Na2HPO4, 0.75 mM NaH2PO4 PH = 7:05,), TE1 × (10 mM Tris-HCL, 1 mM EDTA; PH = 8), Cacl2 (2.5 Mm) and Buffered Water ( 2.5 mM HEPES PH = 7.5) on a vortex [18]. In the mentioned method, First, 4000 HEK 293T cells were seeded in a T75 flask with DMEM culture media (Gibco, USA) and 10% FBS (Gibco, USA) in standard cell growth condition (at 37 °C and 5% CO_2_). Two hours before transfection, the culture media was changed with DMEM containing 4% FBS and stored at 37 °C and 5% CO_2_ for two hours. Then, the mentioned calcium phosphate reagents were blended with helper and lentiviral vectors (psPAX2, PMD2, and PCDH-TK) in a Falcon 50 mL tube on the vortex (this condition provided suitable O2 for calcium phosphate reaction). Thus, 912 µl buffered water, 33 µl TE 1X, 52.5 µg plasmids, and 105 µl CaCl2 (2.5 M) were added and mixed in a Falcon 50 mL tube. Then, 1050 µl of HBSS (2x) was slowly added to the Falcon in a dropwise manner, under agitation and vortexing. Finally, after incubation for 5–15 min, it was transferred to a T-75 flask, which was moved backward and forward as well as side to side homogenously. The transfection efficiency was determined with fluorescence microscopy at 16 h after transfection (Fig. 1A).

**Fig. 2:**
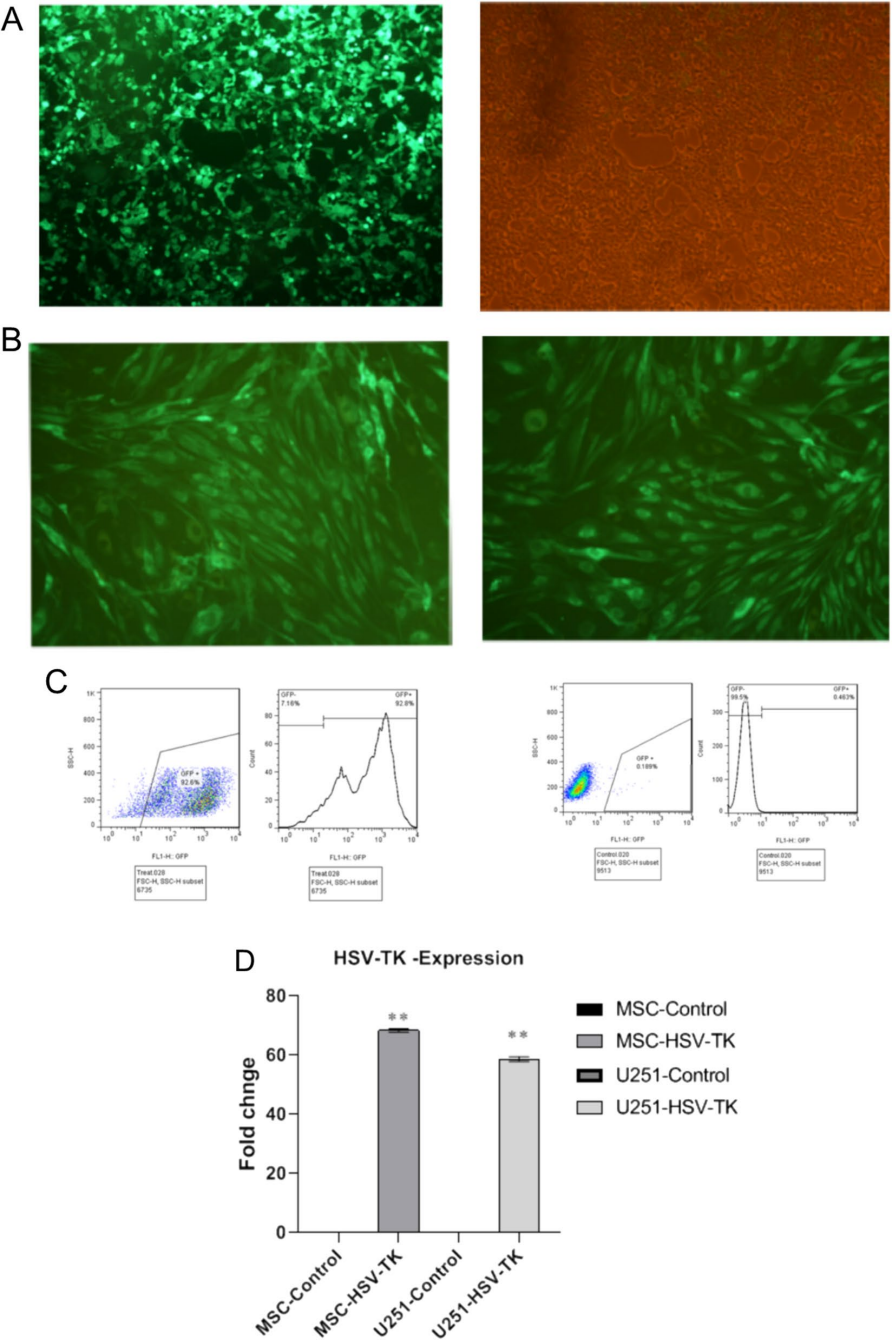
HEK-293T, as packaging host, were co-transfected by psPAX2, pMD2.G, and PCDH-TK with the ratio of 2:1:2 and calcium phosphate reagent (**A**). Proliferation rate of high efficiency transduced MSCs based on fluorescent microscopy after 72 h (**B**). Flow cytometry assay confirmed the population of GFP + cells (92%) (**C**). The relative HSV-TK mRNA overexpression level in transduced MSCs and U-251 cells compared to control is demonstrated via bar chart (** p < 0.01)

**Fig. 3:**
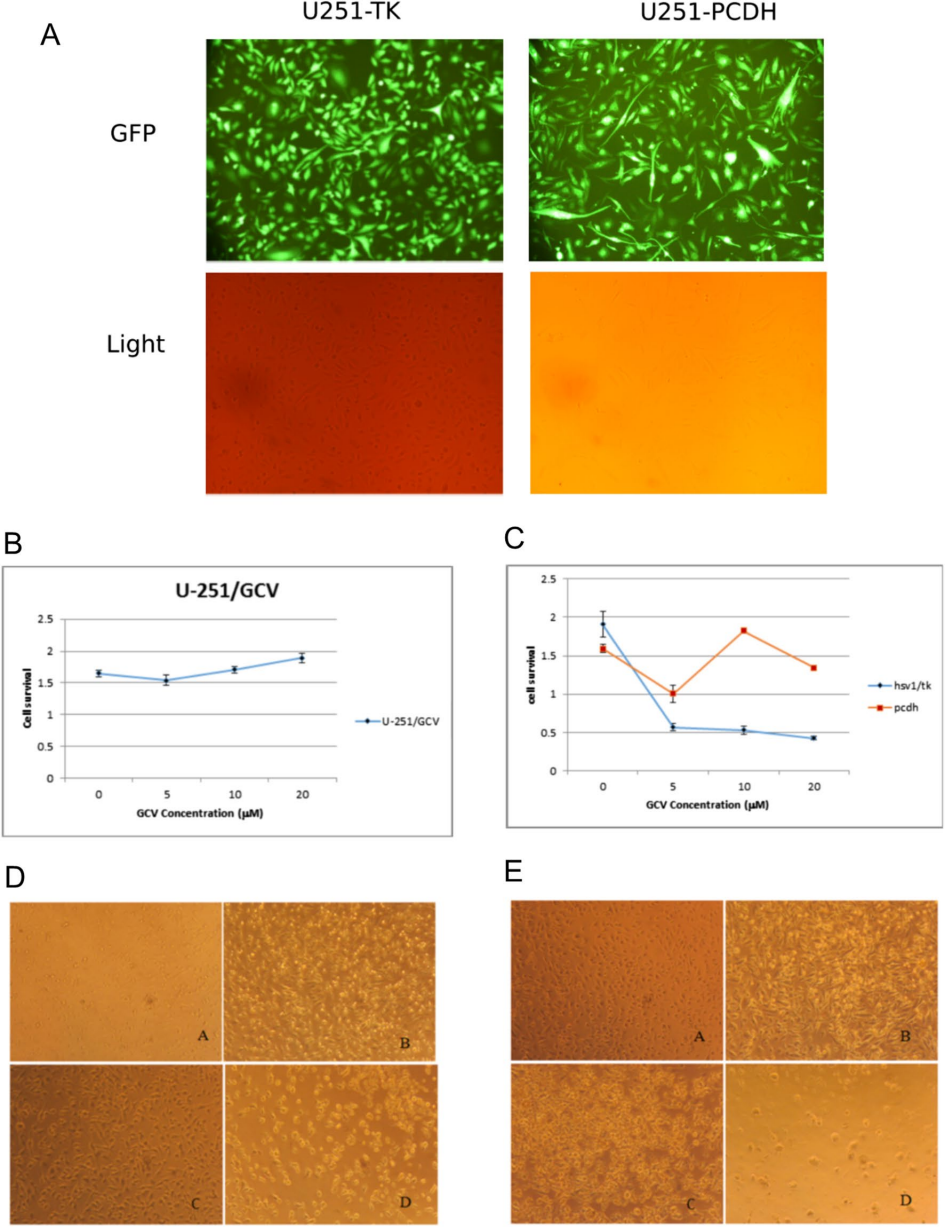
The high efficiency transduced U-251/PCDH (transfected with the plasmid without the HSV-TK gene) and U-251/HSV-TK cells (more than 80% of population) observed using fluorescent microscopy (**A**). The viability of U-251 in serial dilution rate of ganciclovir (GCV) concentration was determined with MTT assay (* p < 0.05) (**B**). The relative cell survival rates of U-251/TK and U-251/PCDH cells treated in a range of GCV concentration for 96 h (* P  < 0.05) (**C**). U-251/PCDH (**D**) and U-251/HSV-TK (**E**) in 0 µg/mL (**A**), 5 µg/mL (**B**), 10 µg/mL (**C**), and 20 µg/mL (**D**) of GCV

After confirming the high quality of transfection, the supernatant was isolated every 12 h for 3 days and concentrated using ultracentrifuge at 47,000 x g for 2 h at 4 °C. The TK expression in ADSCs was induced through their culture in DMEM-F12 medium and transduction with pCDH-TK lentivirus. A total of 3 × 10^5^ cells were transferred to a T-25 flask and transduced with a multiplicity of infection (MOI) of 40 TU/cell. Moreover, cells transduced with pCDH-TK were selected using puromycin 2 µg/mL, and the efficiency of transduction was determined with fluorescent microscopy and flow cytometry (Fig. 2B, C). The U-251 cell line was also transduced and purified with concentrated mock viruses (pCDH) and pCDH-TK viruses (Fig. 3A).

To investigate the HSV-TK expression, the total RNA was extracted from 5 × 10^5^ transduced and control (un-transduced) MSCs and U-251 cell line 72 h after transduction using Trizol buffer (Thermo Fisher Scientific), according to manufacturer’s protocol. Then, the cDNA was synthesized from total RNA using reverse transcriptase enzyme -M-MuLV Reverse Transcriptase enzyme (Thermo Fisher Scientific), and random hexamer primer (for total mRNA). Finally, real-time PCR was performed in triplicate on the step one real-time PCR system (ABI, USA) using SYBR Green master mix2X (Ampliqon, Odense M, Denmark) as well as HSV-TK primers (forward primer: 5´-AATCGCGAACATCTACAC-3´; reverse primer: 5´-CCAGCATAGCCAGGTCA-3´) and HPRT1 primers as an internal control and normalizer (forward primer: 5´-CCT GGC GTC GTG ATT AGT G-3´, reverse primer: 5´-TCA GTC CTG TCC ATA ATT AGT CC-3´). The 2−∆∆CT method and graph pad prism with sample T-test were used for statistical analysis (Fig. 2D).

The effect of HSV-TK gene expression on the U-251 glioblastoma cell line in the presence of ganciclovir was measured by MTT assay. First, the resistance of the U-251 cell line against ganciclovir was determined by exposing this cell line to different concentrations of ganciclovir (0, 5, 10, and 20 µM) and investigating cell viability with MTT assay. Second, 5 × 10^3^ of each transduced cell were seeded in the 96-well plate with DMEM culture medium (Gibco, USA) and 10% FBS (Gibco, USA) in triplicate. Then, the control (U-251-PCDH) and U-251-TK cells were treated with different concentrations of ganciclovir (0, 5, 10, and 20 µM) for 96 h under standard cell culture conditions. After that, the survival rate was calculated using the MTT assay (Fig. 3).

To prepare transduced MSCs for injection, following the purification using puromycin, cells were washed three times with normal saline and prepared for injection. Potential endotoxin contamination was prevented by using EndoFree Plasmid Qiagen Maxi Kit (Cat No./ID: 12,362) for plasmid extraction. Potential contamination with mycoplasma was evaluated using PCR-based methods with universal mycoplasma primers (forward primer: 5´-GGC GAA TGG GTG AGT AAC ACG-3´ and reverse primer: 5´-CGG ATA ACGC TTG CGA CCT ATG-3´) for detecting all mycoplasma species before and after the antibiotic usage. No mycoplasma infection was observed.

### Procedures

Prior to study intervention and tumor recurrence, all patients had received standard treatment for newly diagnosed GBM, which was surgery followed by standard Stupp protocol, including radiation therapy with a total dose of 60 Gy given in 2 Gy daily fractions over a period of 6 weeks and chemotherapy with temozolomide 75 mg/m^2^/day. The standard chemoradiotherapy regimen was followed by adjuvant chemotherapy, including six cycles of temozolomide (150 mg/m^2^/day for 5 days in the first cycle and 200 mg/m^2^/day for 5 days every 28-day cycle). However, given the exclusion criteria of the study, no patient had undergone any treatment, including surgery for recurrent GBM before the suicide gene therapy.

The study intervention mainly consisted of two steps, including direct injection of HSV-carrying ADSCs into the tumor and prodrug administration. The ADSC administration was performed using frameless stereotaxy and neuronavigation system. Following the localization of the tumor by preoperative stereotactic MR imaging, intracerebral stereotactic injection to the cavity was performed under general anesthesia. After the needle reached the central portion of the tumor, through using a guiding cannula, 1 mL of ADSC-containing suspension was manually injected over 1 min. The needle was kept in place for two minutes after the injection. Given the study design, it included three cohorts of patients, and based on the cohort number, different numbers of HSV-TK-carrying ADSCs were administered. Cohort 1 (n = 3) received 2.5 × 10^5^ ADSCs, cohort 2 (n = 3) received 5 × 10^5^ ADSCs, and cohort 3 (n = 3) received 10 × 10^5^ ADSCs. Following the stereotactic injection, patients underwent standard postoperative care. Three days after the cell injection, the prodrug administration regimen, including intravenous (IV) infusion of ganciclovir 5 mg/kg over one hour and every 12 h for 14 days, was initiated. During the postoperative course, all patients were given low-dose maintenance steroids. Patients were discharged on postoperative day 17.

### Outcomes

Since the main aim of this study was to evaluate the safety of suicide gene therapy, a data and safety monitoring board (DSMB), including different experts in relevant fields was established. Each patient was followed up for 24 months after the suicide gene therapy and the overall study duration was 26 months. The primary outcome measure of the present study was the safety profile of suicide gene therapy using allogeneic HSV-TK carrying ADSCs in patients with recurrent GBM, which was defined as the absence of dose-limiting toxicity (DLT). During this investigation, AEs were monitored and graded according to Common Terminology Criteria for Adverse Events (CTCAE) version 4. DLT was defined as any grade III or more non-hematological or grade IV or more hematological AEs associated with study intervention according to the DSMB decision and occurring within 4 weeks following the stereotactic cell injection. Based on the 3 + 3 design of the study, the maximum tolerated dose (MTD) was defined as the highest dose at which no more than 0/3 or 1/6 patients experienced a DLT. If one DLT was observed in any cohort, three more patients were recruited to that dosing cohort. Safety monitoring was performed using serial physical examinations, electrocardiography, and complete laboratory investigations, including serum biochemistry, hematological, coagulation, renal, hepatic, cardiac, and metabolic profiles. In addition to baseline, all the aforementioned assessments were performed on a daily basis during the first 17 days after the surgery and monthly afterward until 3 months postoperatively. Further, all patients were informed about all the potential treatment-related AEs before discharge, and also weekly contacted through regular telephone calls. As part of safety evaluation and potential early effects of suicide gene therapy, MR imaging was also performed at baseline, 3-day (before prodrug administration and after cell injection), and 17-day (before discharge) after the injection.

In addition, potential effects of suicide gene therapy using HSV-TK-carrying ADSCs on the tumor were investigated. Secondary outcome measures for potential efficacy assessment included CET and fluid-attenuated inversion recovery (FLAIR) volumes, progression-free survival (PFS), and overall survival (OS). Quantitative volumetric analysis of changes in CET and FLAIR volumes following the study intervention and over time was performed using semi-automated segmentation method. Moreover, tumor response and progression was evaluated based on Response Assessment in Neuro-Oncology (RANO) response criteria in the present study [19]. Therefore, for evaluation of the tumor response and progression status, in addition to baseline and early time points (3- and 17-day after the injection), MR imaging was carried out every 2 months after the cell injection. PFS was defined as the time from stereotactic cell injection to the first disease progression based on RANO response criteria, and OS was defined as the time from stereotactic cell injection to death. Furthermore, changes in level of serum cytokines, peripheral blood cells and CD markers were also evaluated using enzyme-linked immunosorbent assay (ELISA, Abcam) and flow cytometry, respectively. Concerning this, blood samples were drawn at baseline, day 3, day 17, and day 60 after the cell injection.

### Statistical analysis

Based on 3 + 3 design of the study, all patients in this study were included in statistical analysis (n = 12). In this study, quantitative data were presented as mean ± standard deviation or median with interquartile range and qualitative data were expressed as frequency and percentage. Kaplan-Meier curves were used to demonstrate OS and PFS with 95% confidence interval (CI) for the total study population, as well as based on MGMT methylation. To compare continuous variables between different time points and baseline, Wilcoxon signed-rank test was used. P < 0.05 were considered statistically significant. All statistical analyses in the present study were performed using “R” version 3.6.3.

## Results

### Patient characteristic

**Fig. 4:**
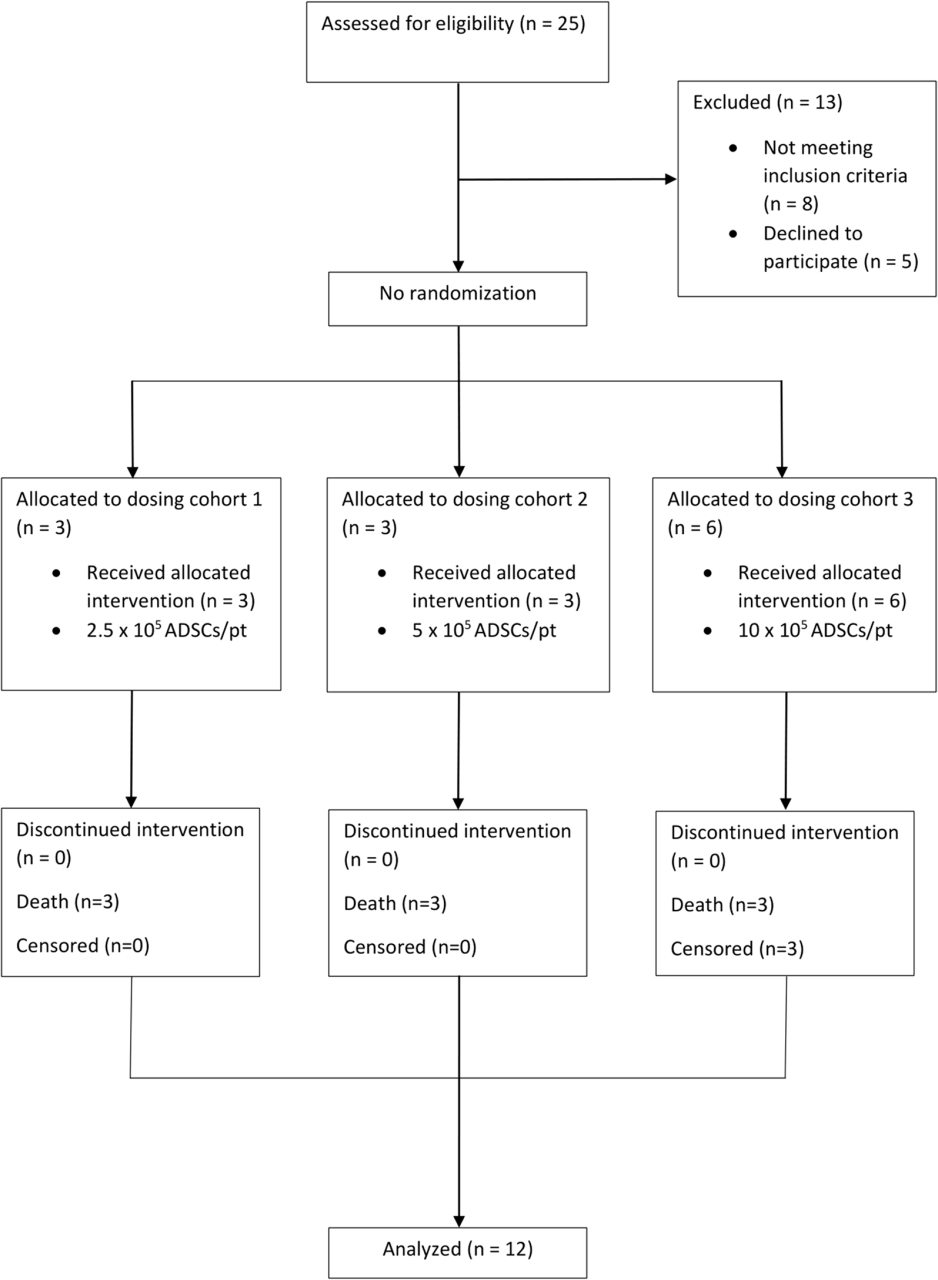
Study flow diagram

Between October 2020 and March 2021, a total of 25 patients with recurrent GBM were assessed for eligibility. Among them, a total of 8 patients did not meet the study criteria, and 5 refused to receive the study intervention. Therefore, a total of 12 patients were included and assigned to study cohorts. No dropout occurred throughout the study period, and all patients received the suicide gene therapy based on the study protocol and their cohort number (Fig. 4).

**Fig. 5:**
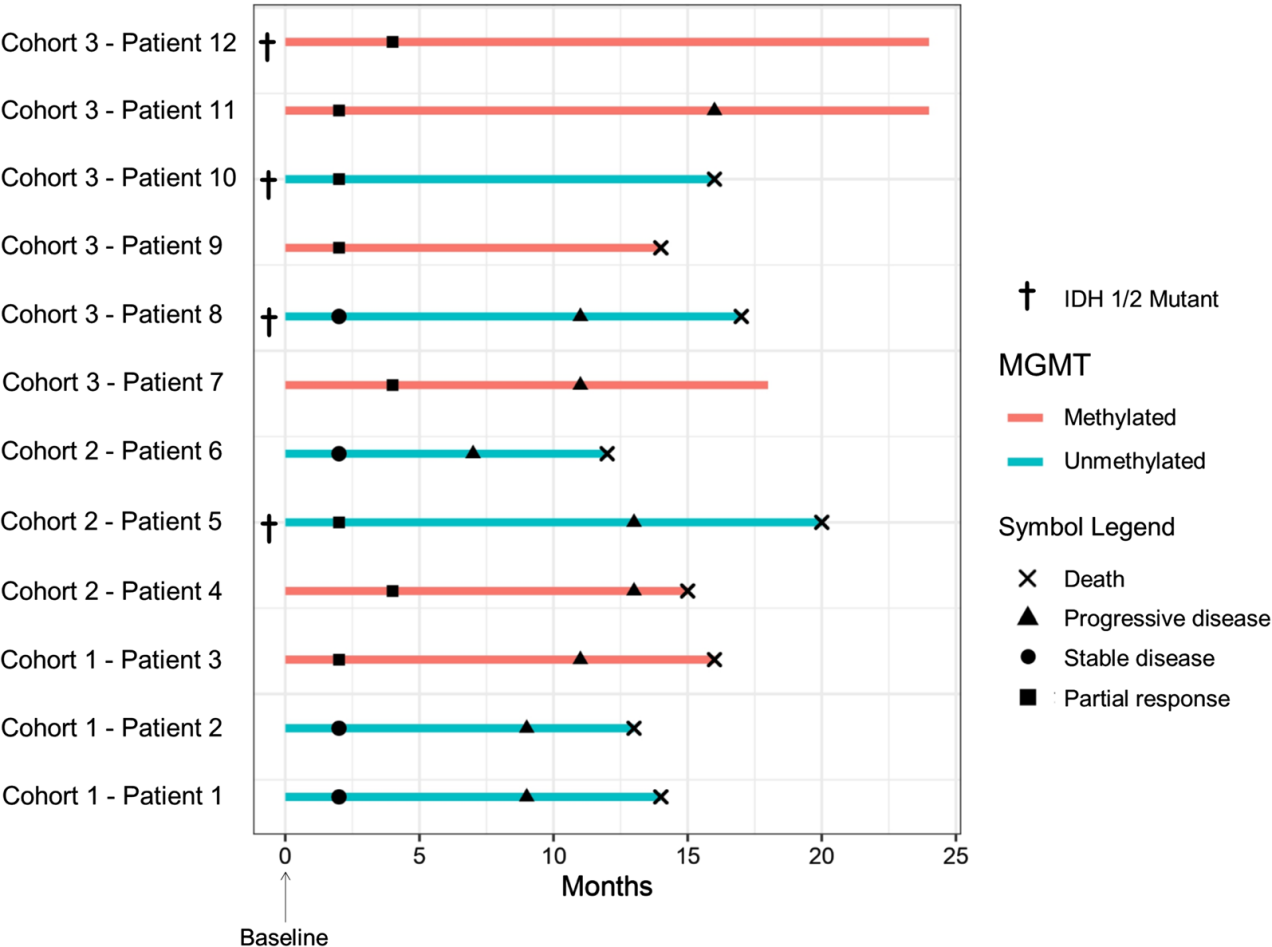
Swimmer’s plot demonstrating the follow-up course of each patient individually with clinical and radiological outcomes after suicide gene therapy

All patients (100%) in the present study had recurrent GBM based on histopathological evaluation of biopsy specimens obtained during the stereotactic surgery and before the injection of study product. A total of 8 patients were male (66.7%) and the mean age of the study population was 56.8 ± 5.8 years. In terms of molecular tumor profile, 4 patients (33.3%) had IDH1-mutated tumors and methylation of MGMT gene promoter was observed in 6 patients (50.0%). Regarding the site of recurrence, 5 (41.7%), 4 (33.3%), and 1 (8.3%) tumors were located in frontal, temporal, and parietal lobes, respectively. In addition, two tumors had temporoparietal (8.3%) and frontotemporal (8.3%) location. Median follow-up time in this study was 16 (IQR, 14–18.5) months. Table 1 demonstrates demographic, clinical, and surgical patient characteristics. Figure 5 includes a swimmer’s plot illustrating the follow-up course of each patient over the study period with clinical and radiological outcomes.

**Table 1:** Demographic, clinical, and surgical characteristics of patients

Characteristic	Overall (n = 12)	Cohort 1 (n = 3)	Cohort 2 (n = 3)	Cohort 3 (n = 6)
Age (years)	56.8 (5.8)	60.7 (5.1)	54.3 (4.0)	56.0 (6.7)
Sex (male)—no. (%)	8 (66.7%)	2 (66.7%)	3 (100.0%)	3 (50.0%)
KPS—no. (%)				
70	1 (8.3%)	0 (0.0%)	1 (33.3%)	0 (0.0%)
80	6 (50.0%)	2 (66.7%)	1 (33.3%)	3 (50.0%)
90	5 (41.7%)	1 (33.3%)	1 (33.3%)	3 (50.0%)
MGMT promoter—no. (%)				
Methylated	6 (50.0%)	2 (66.7%)	1 (33.3%)	3 (50.0%)
Unmethylated	6 (50.0%)	1 (33.3%)	2 (66.7%)	3 (50.0%)
IDH1/2—no. (%)				
Mutant	4 (33.3%)	1 (33.3%)	1 (33.3%)	2 (33.3%)
Wild-type	8 (66.7%)	2 (66.7%)	2 (66.7%)	4 (66.7%)
Time elapsed since the last radiotherapy session (weeks)	66.5 (12.1)	56.7 (8.1)	73.3 (10.3)	68 (12.9)
Median follow-up (months, IQR)	17 (16–18)	13 (12.5–14.5)	16 (16–17)	18 (18–18)
Extent of resection in the last surgery—no. (%)				
GTR	9 (75.0%)	2 (66.7%)	2 (66.7%)	5 (83.3%)
IR	2 (16.7%)	1 (33.3%)	1 (33.3%)	0 (0.0%)
Biopsy	1 (8.3%)	0 (0.0%)	0 (0.0%)	1 (16.7%)
Chemotherapeutic agent—no. (%)				
Temozolomide	12 (100.0%)	3 (100.0%)	3 (100.0%)	6 (100.0%)
Bevacizumab	4 (33.3%)	1 (33.3%)	1 (33.3%)	2 (33.3%)
Lomustine	1 (8.3%)	0 (0.0%)	0 (0.0%)	1 (33.3%)
Anatomical location—no. (%)				
Frontal	5 (41.7%)	2 (66.7%)	1 (33.3%)	2 (33.3%)
Temporal	4 (33.3%)	1 (33.3%)	1 (33.3%)	2 (33.3%)
Parietal	1 (8.3%)	0 (0.0%)	1 (33.3%)	0 (0.0%)
Frontotemporal	1 (8.3%)	0 (0.0%)	0 (0.0%)	1 (16.7%)
Temporoparietal	1 (8.3%)	0 (0.0%)	0 (0.0%)	1 (16.7%)

*KPS* Karnofsky performance scale, *GTR* gross total resection, *IQR* interquartile range, *IR* incomplete resection

### Primary outcome measure

During the study period, a total of 62 AEs was observed. Merely two (3.2%) cases of superficial surgical site infection (grade II based on CTCAE version 4) and wound dehiscence (grade I) in cohort 2 were attributable to the study intervention (surgical procedure), that were treated successfully. The other 60 (96.8%) cases, including 39 grade I (62.9%), 19 grade II (30.6%), and 2 grade III (3.2%) AEs were not related to the study intervention. The observed grade III AEs were not attributable to the study intervention and included one case of urinary tract infection (1.6%) and one case of pneumonia (1.6%). No DLT was observed in any of the study cohorts. The dose escalation was proceeded to the maximum planned study dose (cohort 3, 10 × 10^5^ cells). Table 2 shows all the AEs observed during the study period with their grading based on CTCAE version 4.

**Table 2:** All adverse events observed during the study period

All adverse events—no. (%)	Grade I	Grade II	Grade III
Atrial fibrillation	1 (1.6%)	1 (1.6%)	0 (0%)
Sinus bradycardia	2 (3.2%)	0 (0%)	0 (0%)
Sinus tachycardia	3 (4.8%)	0 (0%)	0 (0%)
Blurred vision	1 (1.6%)	0 (0%)	0 (0%)
Fatigue	2 (3.2%)	3 (4.8%)	0 (0%)
Fever	3 (4.8%)	0 (0%)	0 (0%)
Pneumonia	0 (0%)	0 (0%)	1 (1.6%)
Urinary tract infection	0 (0%)	0 (0%)	1 (1.6%)
Lymphocyte count decreased	2 (3.2%)	1 (1.6%)	0 (0%)
Neutrophil count decreased	1 (1.6%)	2 (3.2%)	0 (0%)
Platelet count decreased	1 (1.6%)	0 (0%)	0 (0%)
Anorexia	2 (3.2%)	2 (3.2%)	0 (0%)
Hypercalcemia	1 (1.6%)	0 (0%)	0 (0%)
Vomiting	3 (4.8%)	0 (0%)	0 (0%)
Nausea	4 (6.5%)	0 (0%)	0 (0%)
Dizziness	1 (1.6%)	1 (1.6%)	0 (0%)
Hypotension	2 (3.2%)	1 (1.6%)	0 (0%)
Headache	2 (3.2%)	2 (3.2%)	0 (0%)
Lethargy	3 (4.8%)	1 (1.6%)	0 (0%)
Seizure	0 (0%)	2 (3.2%)	0 (0%)
Dyspnea	1 (1.6%)	0 (0%)	0 (0%)
Thromboembolic event	1 (1.6%)	2 (3.2%)	0 (0%)
Diarrhea	1 (1.6%)	0 (0%)	0 (0%)
Hyperglycemia	2 (3.2%)	1 (1.6%)	0 (0%)
Intervention-related adverse events—no. (%)			
Wound dehiscence	1 (1.6%)	0 (0%)	0 (0%)
Wound infection	0 (0%)	1 (1.6%)	0 (0%)

### Secondary outcome measures

**Fig. 6:**
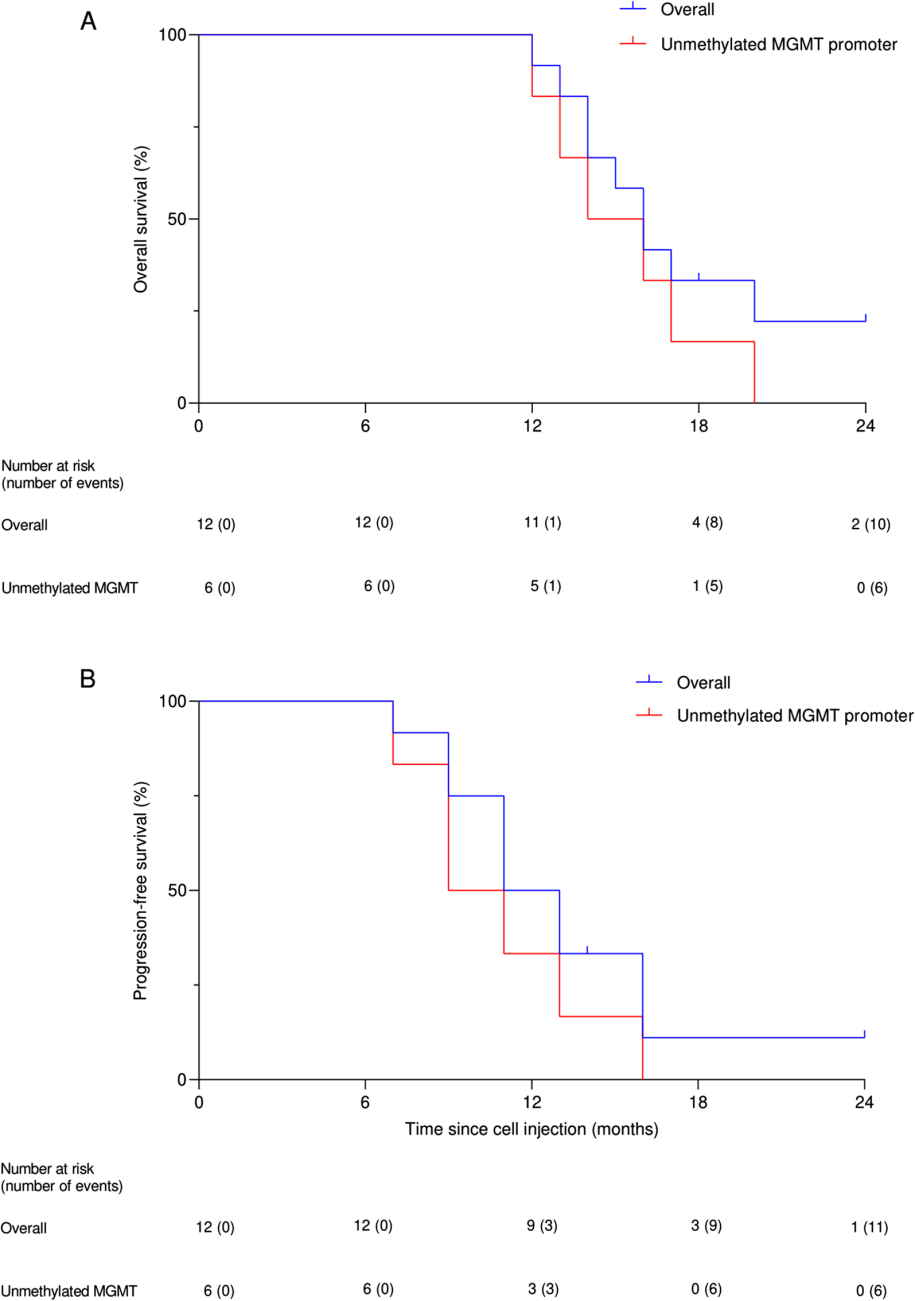
Kaplan-Meier curve for both overall survival (OS) (**A**) and progression-free survival (PFS) (**B**) in all patients and those with unmethylated MGMT promoter undergoing suicide gene therapy

Best response assessment showed partial response and stable disease in 8 (66.7%) and 4 (33.3%) patients, respectively, based on RANO criteria. Two patients in cohort one, one patient in cohort two, and one patient in cohort three had stable disease based on best response assessment. Others showed partial response as the best response. During the study period (at the time of database lock), nine (75.0%) of 12 patients had died, and eleven (91.7%) of 12 patients had shown tumor progression. The median OS was 16.0 months (95% CI 14.3–17.7) and the median PFS was 11.0 months (95% CI 8.3–13.7) in the present study. Patients with unmethylated MGMT promoter had a median OS of 14 months (95% CI 10.4–17.6) and a median PFS of 9.0 months (95% CI 5.8–12.2). Figure 6 demonstrates the Kaplan-Meier curves for both PFS and OS in the study population.

**Fig. 7:**
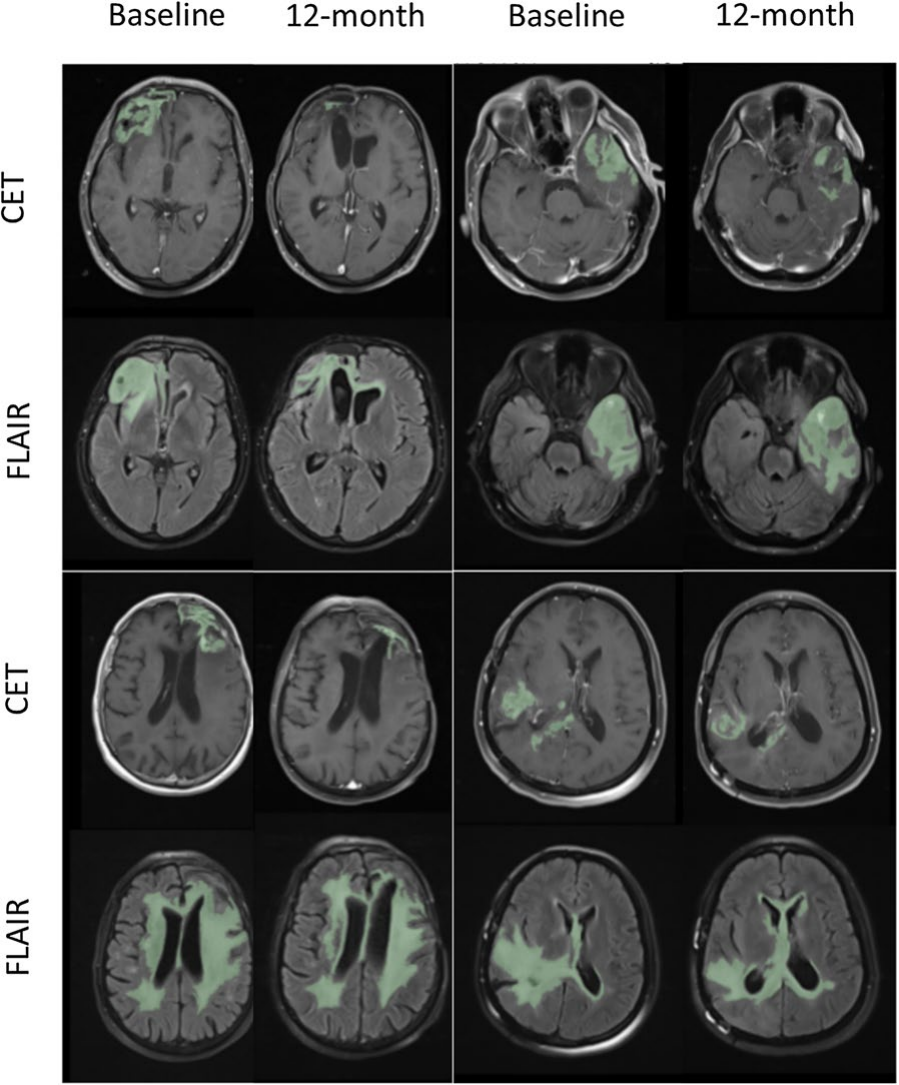
MR images, including post-gadolinium T1 weighted images and fluid attenuated inversion recovery (FLAIR) at baseline and 12-month after the suicide gene therapy using ADSC vehicles in four patients showing changes in contrast-enhanced tumor (CET) and FLAIR volumes

**Fig. 8:**
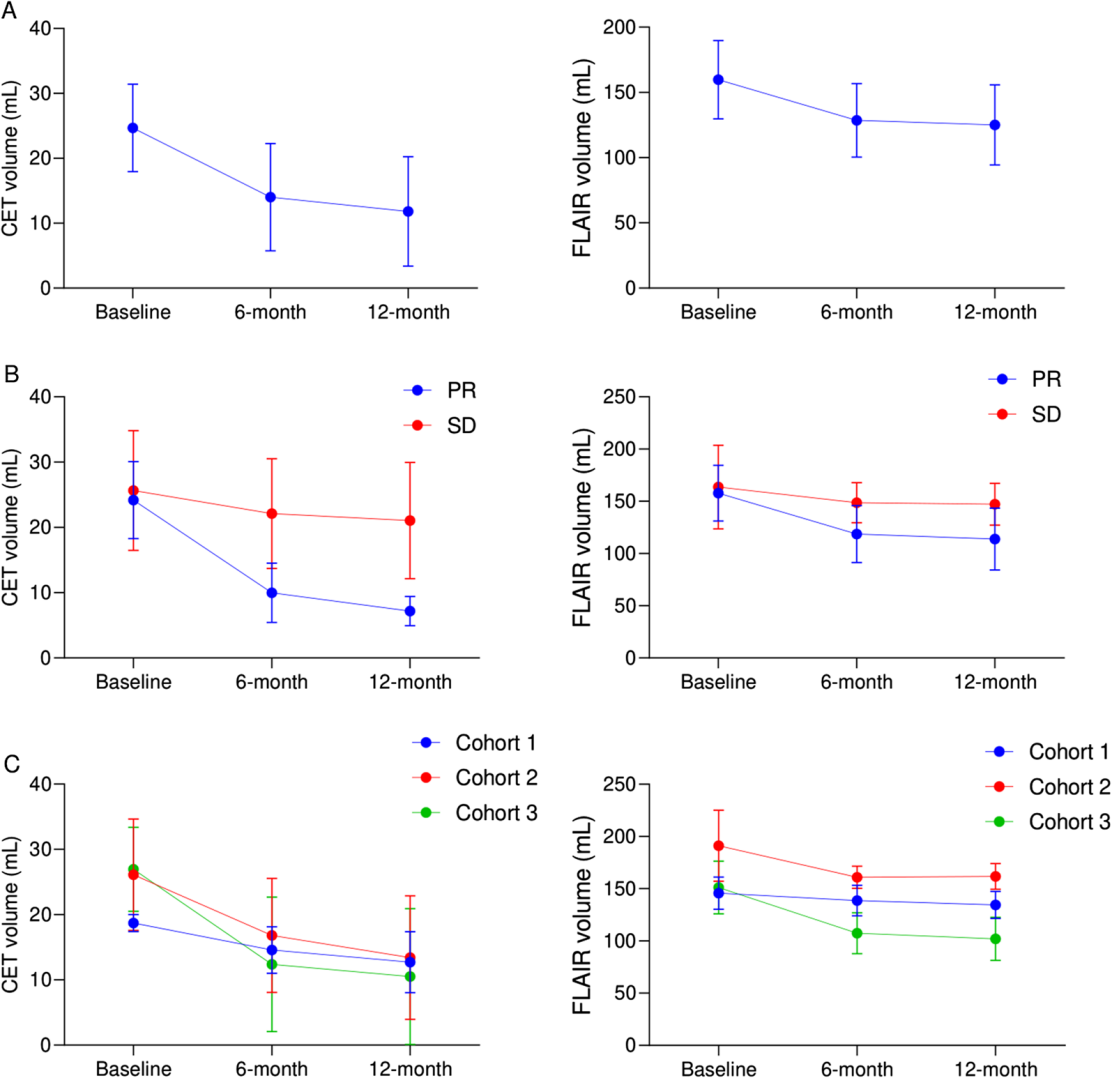
Volumetric analysis of changes in contrast-enhanced tumor (CET) and fluid attenuated inversion recovery (FLAIR) volumes in patients over the study period. **A**. Data for the total study population (n = 12) based on tumor response (RANO criteria) (**B**), and based on dosing cohort (**C**). Data are represented as mean ± SD. *PR* partial response, *SD* stable disease

Based on volumetric analysis of changes in tumor volume, mean CET volume was significantly reduced at 6- (p = 0.002) and 12-month (p = 0.002) after the cell injection compared with the baseline (Figs. 7 and 8). In addition, mean FLAIR volume significantly decreased at 6-(p = 0.005) and 12-month (p = 0.005) after the injection compared with the baseline (Table 3). Figure 8 illustrates volumetric analyses on changes in CET and FLAIR volumes in study population (n = 12) as well as changes based on tumor response and dosing cohort.

**Table 3:** Quantitative volumetric analysis of changes in contrast-enhanced tumor and fluid attenuated inversion recovery volumes over the study period

		Baseline	6-month	12-month
CET (mL)	Cohort 1	18.7 (1.3)	14.6 (3.6)	12.7 (4.7)
	Cohort 2	26.1 (8.5)	16.8 (8.7)	13.4 (9.5)
	Cohort 3	27 (6.4)	12.4 (10.3)	10.5 (10.4)
	PR	24.2 (5.9)	10 (4.6)	7.2 (2.3)
	SD	25.7 (9.2)	22.1 (8.4)	21.1 (8.9)
	Overall	24.7 (6.7)	14 (8.3)	11.8 (8.5)
	P-value^†^	–	0.002^*^	0.002^*^
FLAIR (mL)	Cohort 1	145.7 (15.4)	138.7 (14.7)	134.5 (13)
	Cohort 2	191.1 (34)	160.9 (10.7)	161.9 (12.5)
	Cohort 3	151.2 (25.1)	107.5 (19.7)	101.9 (20.8)
	PR	157.8 (26.7)	118.7 (27.3)	113.9 (29.7)
	SD	163.7 (40.1)	148.6 (19.2)	147.3 (20.1)
	Overall	159.8 (30)	128.6 (28.2)	125 (30.7)
	P-value^†^	–	0.005^*^	0.005^*^

*CET* contrast-enhanced tumor *FLAIR* fluid attenuated inversion recovery, *PR* partial response, *SD* stable disease

* Indicates statistically significant

† Wilcoxon signed-rank test was performed to compare the values between each time point and baseline

**Fig. 9:**
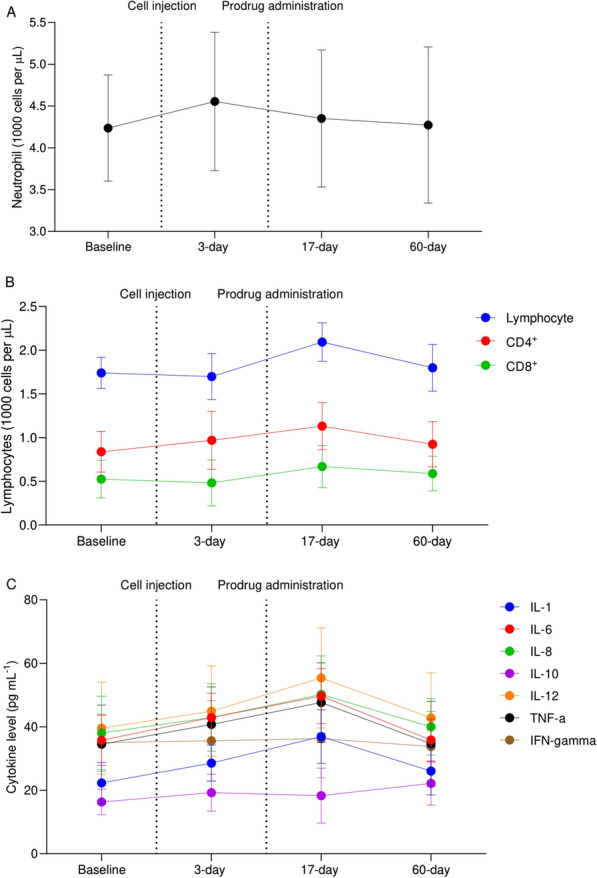
Changes in peripheral blood cells, including neutrophils (**A**) and lymphocytes (**B**) as well as cytokine profile (**C**) over the study period. Data are represented as mean ± SD

Changes in peripheral blood cells and serum cytokine levels were also assessed over the study period in all patients (n = 12). No significant change was observed in absolute neutrophil count at 3—(p = 0.084), 17—(p = 0.530), and 60-day (p = 0.875) postoperatively compared with the baseline. Absolute lymphocyte count, however, peaked at 17-day postoperatively, which was statistically significant compared with the baseline (p = 0.002). Similarly, both CD4^+^ (p = 0.002) and CD8^+^ T cells (p = 0.002) significantly increased at 17-day postoperatively compared with the baseline. Mean absolute lymphocyte (p = 0.028), CD4^+^ (p = 0.013), and CD8^+^ T cell (p = 0.041) counts were also significantly increased at 60-day postoperatively compared with the baseline. Except for IFN-γ (p = 0.478), all other cytokines, including IL-1 (p = 0.002), IL-6 (p = 0.002), IL-8 (p = 0.002), IL-12 (p = 0.002), and TNF-α (p = 0.002) significantly increased at 17-day after the cell injection compared with the baseline. Figure 9 illustrates changes in cytokine profile and peripheral blood cells over the study period.

## Discussion

The present first-in-human phase I clinical trial demonstrated several significant findings regarding the use of a novel allogeneic stem cell-mediated suicide gene therapy protocol in patients with recurrent GBM. The suicide gene therapy protocol, including stereotactic injection of allogeneic ADSC gene delivery vehicles was safe in patients with recurrent GBM and no early- or late-onset complication was observed over the study period. Additionally, among 12 patients, 8 showed partial response and others had stable disease as the best response. Volumetric analysis also revealed significant reductions in both CET and FLAIR volumes that were durable for 12 months postoperatively. Further, specific changes in cytokine profile and peripheral blood cells were noted following this gene therapy protocol.

This study found that the present novel suicide gene therapy protocol using allogeneic ADSC gene vehicles carrying HSV-TK gene was safe without any DLT in patients with recurrent GBM. Prior to this investigation, initial early-phase clinical trials and also larger investigations had demonstrated the safety profile of suicide gene therapy using viral vectors, such as retrovirus and adenovirus in patients with GBM [20–22]. Regarding the stem cell-mediated suicide gene therapy, however, to date, our previous study has been the only one evaluating the safety of suicide gene therapy using autologous bone marrow-derived mesenchymal stem cells carrying HSV-TK gene [17]. In that study, a total of 11 AEs with grades I and II were observed out of which merely two cases of fever and two cases of headache were probably associated with the study intervention. In addition to suicide gene therapy, a recent phase I clinical trial showed that intratumoral injection of neural stem cell vehicles delivering engineered oncolytic adenoviruses was safe in patients with newly diagnosed high-grade glioma [23]. Except for one case of viral meningitis (grade III), which was due to intraventricular injection and one case of subdural fluid collection (grade II), other observed AEs were not related to the study intervention. In the present investigation, the only AEs attributable to the gene therapy protocol were two cases of grade II superficial surgical site infection and grade I wound dehiscence, which was mainly caused by the surgical procedure and not the study products. No other AE was related to the study protocol and all AEs were successfully managed.

Furthermore, this novel protocol showed significant potential for improving both PFS and OS in patients with recurrent GBM. According to prior findings, patients with recurrent glioma have a median OS and PFS of 4.93 (95% CI 4.37–5.48) and 1.80 months (95% CI 1.56–2.03), respectively [24]. In the present study, median PFS and OS were 9.0 (95% CI 5.8–12.2) and 16.0 months (95% CI 14.3–17.7), respectively. Therefore, although this study is underpowered for efficacy evaluation, based on results, it seems that suicide gene therapy using the ADSC gene delivery system could significantly improve the survival outcomes of patients with recurrent GBM. Among 12 patients in this study, 8 showed partial response and 4 had stable disease without any other concurrent treatment for recurrent GBM, such as surgery, radiation, or chemotherapy and with merely a single intratumoral stereotactic injection of allogeneic ADSCs carrying HSV-TK gene followed by prodrug administration. By contrast, initial phase III clinical trials with large sample sizes failed to demonstrate any superior efficacy for HSV-TK-mediated suicide gene therapy using viral vectors in comparison with control group [20, 21]. These results are best explained by limitations associated with viral vectors, such as low transduction efficiency in retrovirus-mediated or lack of long-term transgene expression in adenovirus-mediated suicide gene therapy [20–22, 25–27]. Cell-based vectors, however, show several capabilities, such as tumor-tropic migration and homing capacity, which lead to their integration into the tumor microenvironment [4]. In the recent clinical trial performed by Fares et al., which evaluated the use of neural stem cells as a delivery system for oncolytic adenovirus gene therapy, among 12 patients with newly diagnosed GBM, only one patient showed partial response [23]. The median PFS and OS were 9.1 and 18.4 months, respectively. A number of preclinical studies have demonstrated significant cytotoxic efficacy for suicide gene therapy using MSCs and ADSCs expressing HSV-TK in animal models of GBM through various mechanisms, such as “bystander effect”, close association with tumor microvasculature, changing the protein cargo, and inducing a local immune reaction [7–11]. However, to date, except for the present investigation, no clinical study has evaluated the use of allogeneic ADSC carrying HSV-TK as vehicles for suicide gene therapy. Future large high-quality randomized controlled trials are highly needed to compare the efficacy of this novel gene therapy protocol with standard treatment.

In addition, based on MR images, in comparison with the baseline, both CET and FLAIR volumes showed a substantial reduction at 6- and 12-months postoperatively. This long-term response observed in patients with recurrent GBM might be attributable to the cytotoxic effects of suicide gene therapy using ADSC gene vehicles similar to preclinical results indicating the role of “bystander effect” in this therapeutic strategy using ADSCs [7, 8]. Besides, there are both preclinical and clinical reports showing that in addition to the “bystander effect,” anti-tumor local immune response with infiltration of T cells and NK cells could also be another mechanism involved in suicide gene therapy [15, 22, 28–30]. The latter mechanism is also highly suggested by the long-term observed radiological response (reductions on volumetric analysis 12 months after the injection) in patients. Nevertheless, there is no prior clinical study evaluating changes in tumor volume over time in order to make a comparison. Moreover, due mainly to ethical considerations we were incapable of performing biopsy to evaluate the histopathological changes in the tumor. Therefore, since our findings are reported for the first time in human, future preclinical and clinical investigations are highly needed to shed light on potential mechanisms behind these observations.

Immunological profile, including both peripheral blood cells and cytokines showed marked changes over the study period. Absolute lymphocyte, CD4^+^, and CD8^+^ counts peaked at 17-day after the injection and then decreased at 2-month postoperatively, yet mean values were still greater than baseline. Most cytokines also demonstrated a substantial increase at 17-day postoperatively compared with the baseline. In aggregate, the aforementioned changes in immunological profile suggested a remarkable immune response following the use of prodrug at 17-day postoperatively. Similarly, Fares et al. found a significant increase in the number of lymphocytes, CD4^+^, and CD8^+^ 2 weeks following the gene therapy [23]. Additionally, they showed that active CD8^+^ T cells infiltrated the tumor tissue with an increase in CD8^+^:CD4^+^ ratio and expression of CD69 suggesting recent infiltration and cytotoxic activity. They also reported a significant increase in a number of cytokines 2 weeks after the surgery.

Despite many strengths, the present investigations had some limitations. First, given the main aim of this study, which was safety evaluation and MTD determination, it was performed with one single arm without any control group. Although compared with prior evidence, the present gene therapy protocol led to significant results in terms of both clinical and radiological response in recurrent GBM, future phase II and III clinical trials with larger sample sizes and control arms are highly demanded to allow for comparison between this protocol and standard therapy. Second, all the procedures as well as perioperative care was performed in one center, which might have affected the survival and clinical outcome of the patients. Thus, future multicenter investigations should validate these findings. Third, due mainly to ethical considerations, it was not applicable to perform a second biopsy after the stereotactic injection. Moreover, patients who died in this study had decline the autopsy. Accordingly, histopathological analysis of changes in the tumor microenvironment could not be performed. Hence, future preclinical or clinical evidence in this regard is required to clarify the mechanisms underlying observed radiological response in this study. Fourth, this clinical trial only included patients with recurrent GBM, who did not undergo any treatment modality, such as surgery or chemoradiotherapy for their recurrence. Therefore, it is unknown whether the combination of the present cell-based suicide gene therapy protocol and other treatment modalities leads to a better clinical response. Further, future investigations should evaluate the effects of this gene therapy on outcomes of patients with newly diagnosed GBM.

## Conclusions

The present phase I clinical trial indicated that suicide gene therapy through stereotactic intratumoral injection of allogeneic ADSCs carrying HSV-TK gene followed by prodrug administration is safe in patients with recurrent GBM. Moreover, although this study is underpowered, it seems that the present novel suicide gene therapy protocol could lead to significant improvements in PFS and OS in patients with recurrent GBM. Volumetric analysis of MR images also showed significant reductions in both CET and FLAIR volumes after 12 months compared with the baseline. Changes in peripheral blood cells and cytokines also suggested an immune response, which peaked early after the prodrug administration. Although this novel cell-based suicide gene therapy was safe, future phase II/III clinical trials with adequate power are required to compare its efficacy in improving clinical outcome with control group, including patients who merely receive standard therapy.

## Data Availability

The datasets analyzed during this study are available from the corresponding author on reasonable request.

## Abbreviations

ADSC: Adipose tissue-derived mesenchymal stem cell
AE: Adverse event
BMSC: Bone marrow-derived mesenchymal stem cell
CET: Contrast-enhanced tumor
CI: Confidence interval
CONSORT: Consolidated Standards of Reporting Trials
CTCAE: Common Terminology Criteria for Adverse Events
DLT: Dose-limiting toxicity
DSMB: Data and safety monitoring board
ELISA: Enzyme-linked immunosorbent assay
FLAIR: Fluid-attenuated inversion recovery
GBM: Glioblastoma multiforme
GMP: Good manufacturing practice
HEK: Human embryonic kidney
HSV-TK: Herpes simplex virus thymidine kinase
IV: Intravenous
MOI: Multiplicity of infection
MR: Magnetic resonance
MSC: Mesenchymal stem cell
MTD: Maximum tolerated dose
OS: Overall survival
PFS: Progression-free survival
RANO: Response assessment in neuro-oncology
